# Essential literature for the chiropractic profession: a survey of chiropractic research leaders

**DOI:** 10.1186/2045-709X-21-33

**Published:** 2013-09-27

**Authors:** Barbara A Mansholt, John S Stites, Dustin C Derby, Ron J Boesch, Stacie A Salsbury

**Affiliations:** 1 Technique Department, Palmer College of Chiropractic, 1000 Brady St, Davenport, IA 52803, USA; 2 Community Clinics, Palmer College of Chiropractic, 2001 52nd Ave, Moline, IL 61265, USA; 3 Institutional Planning & Research, Palmer College of Chiropractic, 723 Brady St, Davenport, IA 52803, USA; 4 Palmer Davenport Clinical Affairs, Palmer College of Chiropractic, 1000 Brady St, Davenport, IA 52803, USA; 5 Palmer Center for Chiropractic Research, Palmer College of Chiropractic, 741 Brady St, Davenport, IA 52803, USA

**Keywords:** Evidence-based practice, Evidence-based health care, Chiropractic, Web-based survey, Publications, Professional education

## Abstract

**Background:**

Evidence-based clinical practice (EBCP) is an accepted practice for informed clinical decision making in mainstream health care professions. EBCP augments clinical experience and can have far reaching effects in education, policy, reimbursement and clinical management. The proliferation of published research can be overwhelming—finding a mechanism to identify literature that is essential for practitioners and students is desirable. The purpose of this study was to survey leaders in the chiropractic profession on their opinions of essential literature for doctors of chiropractic, faculty, and students to read or reference.

**Methods:**

Deployment of an IRB exempted survey occurred with 68 academic and research leaders using SurveyMonkey®. Individuals were solicited via e-mail in August of 2011; the study closed in October of 2011.

Collected data were checked for citation accuracy and compiled to determine multiple responses. A secondary analysis assessed the scholarly impact and Internet accessibility of the recommended literature.

**Results:**

Forty-three (43) individuals consented to participate; seventeen (17) contributed at least one article of importance. A total of 41 unique articles were reported. Of the six articles contributed more than once, one article was reported 6 times, and 5 were reported twice.

**Conclusions:**

A manageable list of relevant literature was created. Shortcomings of methods were identified, and improvements for continued implementation are suggested. A wide variety of articles were reported as “essential” knowledge; annual or bi-annual surveys would be helpful for the profession.

## Background

**Figure 1:**
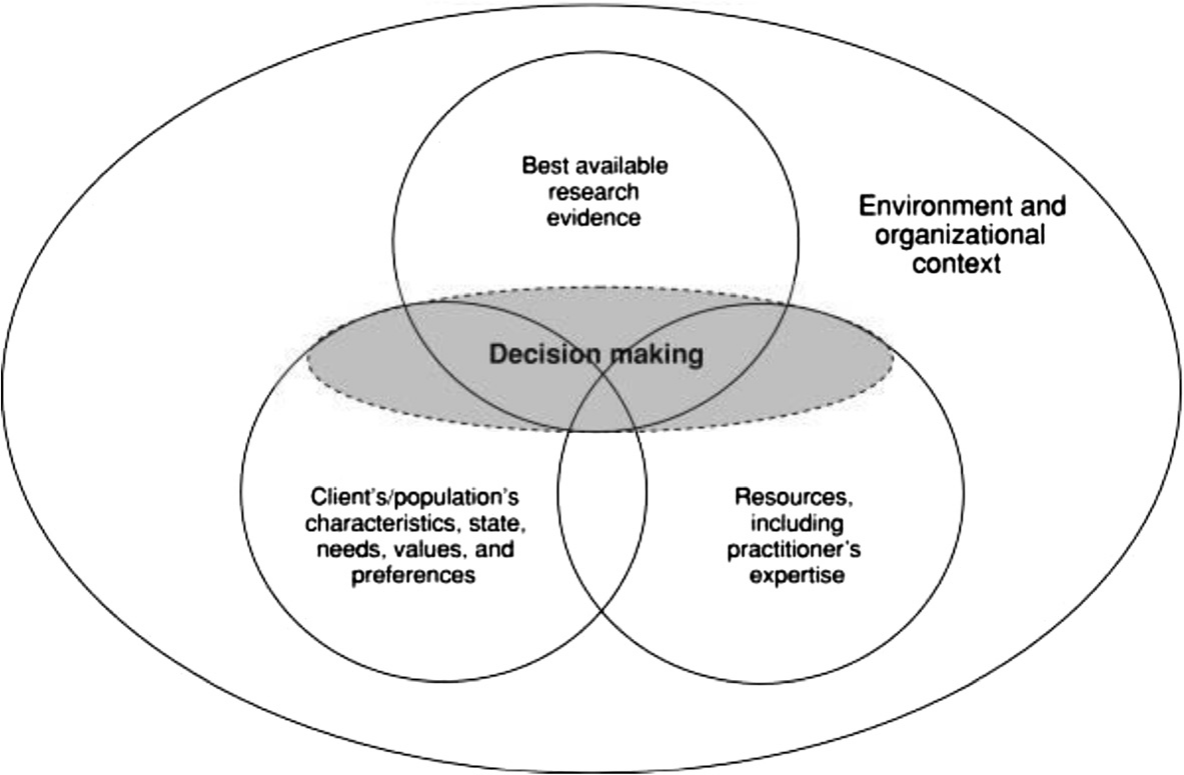
**Evidence based clinical decision making Venn diagram**^**2**^**.**

Evidence based clinical practice (EBCP) may be defined as “the conscientious and judicious use of clinical expertise, patient values, and current best evidence” [1]. Each component of EBCP may assume greater or lesser importance depending upon such factors as the doctor-patient relationship or the patient’s presenting condition and preferences, and assert varying emphases within different healthcare environments or health service delivery contexts [2] (Figure 1). While definitions may vary, evidence within an EBCP framework refers to published, peer-reviewed, scientific research [1]. Thus, EBCP requires that clinicians are knowledgeable of current scientific evidence and best practices based upon published research [3].

The chiropractic profession has maintained its position as the largest alternative or non-traditional approach to healthcare [4-6]. At the same time, the profession seeks wider mainstream acceptance by both the public and its healthcare peers [7-16]. In response, many chiropractic colleges now teach EBCP approaches to chiropractic care [17-20]. Used appropriately, EBCP gives chiropractic professionals of all stages of experience insight beyond their own clinical expertise to guide clinical decision making and helps patients make educated, informed choices [21]. However, recent studies suggest chiropractors and chiropractic students may not yet possess the attitudes, knowledge, reading habits or critical appraisal skills needed to implement EBCP in educational or practice settings [19,22-24].

One issue in EBCP consistently identified by chiropractic students and clinicians is the process of searching, reading, and appraising the scientific literature to apply this evidence to clinical practice [22-26]. At the same time, published research in the chiropractic profession is increasing in quality and quantity at a rapid pace [27-30]. Clinicians in all stages of practice might benefit from compiled resources that identify key studies and other evidence to support chiropractic practice. Chiropractic researchers frequently review and appraise the scholarly literature related to the field of chiropractic and might provide input into which articles would be most important to doctors of chiropractic [28]. Our goal in this study was to compile a list of the most important published research germane to the field of chiropractic—articles every doctor of chiropractic, students, or faculty should read, reference, and put into EBCP.

## Methods

The Palmer College of Chiropractic Institutional Review Board (IRB) Human Protections Administrator exempted this survey from full IRB review, and the study was approved as submitted. The authors compiled a list of potential survey respondents that included the research department directors at 18 chiropractic colleges in North America, editors of 17 chiropractic journals, and respected leaders within the profession based upon their current involvement in research and/or regard as a prominent research author. We identified an initial list of 68 chiropractic research leaders.

We developed a web-based survey using Survey Monkey® software and pre-tested it among the authors. Recipients received the survey link via e-mail and cover letter with a short explanation regarding the purpose and methods of the study. Upon linking to the survey, recipients were informed that no personal benefit would be gained through their response and that their response was anonymous. Checking a box “yes” to advance into the survey served as informed consent and was stated as such in the dialogue box. Survey deployment occurred between August and October 2011. Respondents received up to four automated reminder messages, depending on their response status within the software. Demographic data collected included respondents’ age, gender, highest degree earned, primary academic assignment (e.g., research, faculty, clinician, administration), and area of focus.

The survey used qualitative data collection methods as presenting respondents with an exhaustive list of research studies germane to the field of chiropractic in a checklist format was not feasible. Instead, respondents listed the author, title, journal, and year of an article the respondent considered important and one “that every doctor of chiropractic should read”. Respondents then categorized the article as education, research, health care policy, patient education, or other. Each respondent also wrote a short statement indicating why the recommended article was important for the field. Each respondent could list up to six research studies. Quality control checks assured citation accuracy. Citation recommendations were analyzed thematically according to Bogdan and Biklen’s “subjects” ways of thinking about people and objects” [31]. In other words, we were interested in knowing which objects (e.g., published research) the respondent believed were the most critical for chiropractic practitioners and students (e.g., their thoughts on importance).

We performed a secondary analysis to characterize the scholarly impact and accessibility of each recommended article to assist chiropractic professionals in the process of selecting high-quality evidence from among the scholarly literature. Scholarly impact refers to the relative importance of a publication within its field and across disciplines. Google Scholar was selected as the literature search engine over alternate scientific and academic literature repositories (e.g., PubMed, Index of Chiropractic Literature, Scopus, Web of Science) for its no-cost public access, breadth of coverage across disciplines, ability to search multiple databases simultaneously, embedded links to recent and full-text articles, and literature citation metrics. We estimated scholarly impact by counts of the number of times other researchers had cited each article.

Practitioners’ access to scholarly literature is a known barrier to implementation of EBCP across disciplines with many clinicians reporting little to no access to electronic resources in their workplaces [25,32-34]. Article accessibility was assessed through the ‘All Versions’ feature of Google Scholar. We characterized each article as fully accessible if it was available without cost through open access sources (e.g., PubMed Central [PMC] or the journal/publisher website), limitedly accessible if an article required purchase from the publisher for on-line access, and inaccessible if no link to an on-line source or purchase options was identified.

### Statement on ethics

This study was submitted to the Palmer College of Chiropractic Human Protections Administrator in June of 2011, determined exempt according to 45 CFR 46.101(b)(2), and conducted from August of 2011 through October or 2011. A signed copy of IRB assurance # X2011-2-1-S is available upon request.

## Results

### Response rates and respondent demographics

Although 43 of 68 potential respondents agreed to participate in the study (63%), only 17 respondents contributed at least one article of importance, for a response rate of 25%. Of the final respondents, six were between 41–50 years of age, ten were between 51 and 60, and one was 61 or older. In addition to possessing a clinical doctorate, nine held academic doctorates, six master’s degrees, and twelve bachelor’s degrees. Demographic characteristics of survey respondents are presented in Table 1.

**Table 1:** Respondent demographics of essential literature study

**Characteristic**	**n = 17**
Age	
*41-50*	6
*51-60*	10
*61 or older*	1
Gender	
*Female*	1
*Male*	16
Degrees earned (mark all that apply)	
*BS*	12
*MS*	6
*Clinical doctorate (DC, DO, MD)*	17
*Academic doctorate (PhD or EdD)*	9
Primary involvement in chiropractic profession	
*Research*	8
*Teaching (full-time)*	2
*Faculty Clinician*	1
*Practicing clinician (non-academic)*	3
*Administration*	2
*Other*	1
Area of focus	
*Health care policy*	1
*Patient care*	2
*Education*	3
*Research*	9
*Other*	2

### Article citation data

**Figure 2:**
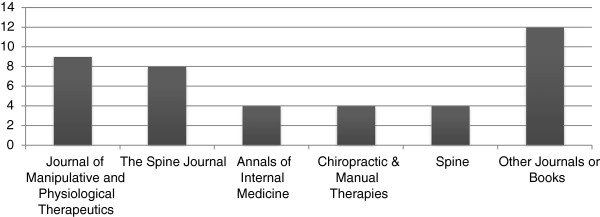
Recommended citations by journal.

**Figure 3:**
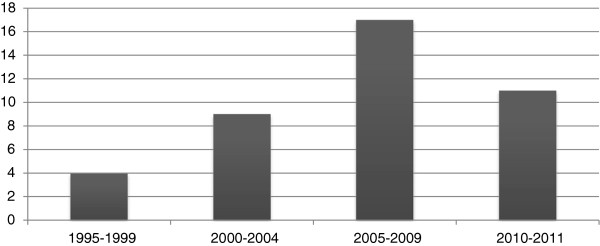
Recommended citations by publication year.

Respondents contributed 50 pieces of evidence (PE). Forty-one PE were mentioned at least once, with six articles mentioned by two or more respondents. Respondents categorized 26 as research, six as education, six as health care policy, and the remaining three as other. Additionally, forty were published within journals and one was a book. Recommended articles were published most often in the *Journal of Manipulative and Physiological Therapeutics* and *The Spine Journal* (Figure 2). Publications years for the recommended PE ranged from 1995 to 2011, with an emphasis on recently published journal articles. Seventeen (17) PE were published in 2005–2009 and 11 PE between 2010–2011 (Figure 3).

Table 2 reports the 41 recommended PE. Citations are listed first by the number of recommendations by survey respondents and then by number of Google Scholar citations. Each PE also includes information about its accessibility. Lastly, a quotation from the recommending respondent is provided demonstrating the importance of the article for chiropractic professionals.

**Table 2:** Essential literature for the chiropractic profession: articles submitted, number of recommendations and justification, Google scholar citation frequency, and accessibility

**Citation**	**Survey votes**	**Google scholar citations**	**Open access available**	***Recommendation quote***
Bronfort G, et al [35]	6	134	PMC	*“This is the most comprehensive systematic review of the literature pertinent to DCs for both musculoskeletal (MSK) and non-MSK conditions.”*
Pickar JG [36]	2	183	Purchase Un/author	*“provides evidence based scientific rationale for the effects of spinal manipulation.”*
Cassidy JD, et al [37]	2	174	PMC	*“This presents the best evidence about the risk of stroke after cervical manipulation.”*
Walker BF, et al [38]	2	32	Purchase	*“This systematic review shows that usual chiropractic care is as effective as many other therapies for acute or sub acute low back pain.”*
Ianuzzi A, et al [39]	2	24	PMC	*“An important paper for mechanisms of action of spinal manipulation.” “Demonstrated intrinsic biomechanical safety of lumbar spinal manipulation.”*
Murphy DR, et al [8]	2	6	Journal	*“This article outlines the need for a primary spine care clinician in the health care system, and highlights the qualifications of DCs to fill this niche.”*
Chou R, et al [40]	1	753	Journal	*“Excellent summary of 7 recommendations related to the diagnosis and treatment of low back pain.”*
Childs JD, et al [41]	1	435	Journal	*“…published by PTs and is cited as the first clinical prediction rule (CPR) for spinal manipulation. Although as DCs we feel “underwhelmed” by the simplistic nature of this CPR, we must recognize that this paper has catapulted the PT profession’s image within the spine care world.”*
Chou R, et al [42]	1	389	Journal	*“clinical practice guidelines for primary care physicians for treatment of low back pain that includes spinal manipulation”*
UK BEAM Trial Team [43]	1	245	PMC	*“largest effectiveness trial to date of benefits of spinal manipulation for low back pain”*
Senstad O, et al [44]	1	209	Purchase	*“Chiropractors need to know that there are side effects to SMT and that not all patients have a positive experience or outcome.”*
Hurwitz EL, et al [45]	1	207	Purchase Un/author	*“The authors assess the evidence base for spinal manipulation and other conservative therapies for the treatment of neck-related disorders.”*
Nelson CF, et al [46]	1	154	Purchase	*“showed long term benefits of manipulation over most commonly used medication for migraine headaches.”*
Olafsdottir E, et al [47]	1	141	Journal	*“This paper demonstrates that the benefit of chiropractic for infantile colic is the same as a placebo.”*
Skyba DA, et al [48]	1	88	PMC	*“This paper presents the best neurophysiological evidence regarding post manipulative analgesia.”*
Haldeman S, et al [49]	1	79	PMC	*“good summary of status of neck pain research relevant to chiropractors; would recommend all papers in that series”*
Dagenais S, et al [50]	1	76	Purchase Un/author	*“important to understand the current evidence and its implication for practice.”*
Legorreta AP, et al [51]	1	59	Journal	*“excellent paper assessing cost benefits to chiropractic care”*
Cramer GD, et al [52]	1	56	Purchase Un/author	*“mechanisms of action of spinal manipulation”*
Haas M, et al [53]	1	50	Purchase Un/author	*“Dose studies of manipulation are extremely important. This one showing that more care [is] better than less…”*
Carroll LJ, et al [54]	1	44	PMC	*“The authors assess the evidence base for spinal manipulation and other conservative therapies for the treatment of neck-related disorders.”*
Bogduk N, et al [55]	1	37	No online access	“This would give chiropractors a clear insight into and synthesis of the evidence available for the treatment and management of neck pain”
Pickar JG, et al [56]	1	37	Purchase	*“important paper for mechanism of action of spinal manipulation”*
Haas M, et al [57]	1	34	Purchase Un/author	*“provides evidence that the frequency of effective manipulative care may not be what is generally thought in clinical practice”*
Reggars JW, et al [58]	1	33	Purchase	*“it demonstrates which joints cavitate in the neck when the head is rotated left or right.”*
Murphy DR, et al [59]	1	32	Journal	*“This paper is the first in a series to present a rationale for and the details of a rational evidence based approach to the management of patients with spinal pain.”*
Bishop PB, et al [60]	1	27	Purchase Un/author	*“demonstrates practical application of EBM to clinical care”*
Little JS, et al [61]	1	26	Purchase	*“demonstrated that vertebral motion unit fixation (aka subluxation) creates abnormal facet joint capsule strain patterns”*
Pickar JG, et al [62]	1	23	PMC	*“demonstrated [that] spinal manipulation induces paraspinal muscle afferent response”*
Herzog W [63]	1	19	Purchase Un/author	*“reviews the current evidence related to adjusting”*
Haas M, et al [30]	1	18	Purchase	*“provides a historical summary”*
Murphy DR, et al [59]	1	15	Journal	*“2nd in the series by Murphy et al. that presents the evidence for the model presented earlier on a chiropractic management approach to spinal pain”*
Ianuzzi A, et al [64]	1	14	Purchase	*“determined unique biomechanics created by spinal manipulation”*
Hartman SE [65]	1	13	PMC Journal	*“helps explain why our belief in clinical effectiveness may not jibe with the research”*
Henderson CN, et al [66]	1	13	Purchase Un/author	*“establishes animal model for studying mechanisms of action of spinal manipulation”*
McGinn T, et al [67]	1	11	PMC	*“teaches CPRs in applying evidence”*
Cramer GD, et al [68]	1	10	Purchase	*“mechanism of action of spinal manipulation”*
Chevan J, et al [69]	1	8	Journal	*“This article written by PTs cites survey research showing that more LBP patients seek DC services (28%) compared to PT services (11%). Their discussion about the disparities between DCs and PTs is quite revealing.”*
Villanueva-Russell Y [14]	1	6	Purchase	*“helps create professional identity”*
Bogduk N [70]	1	3	No online access	*“This article provides the reader with the [simple] tools to determine if a manuscript provides the necessary information to identify the reliability and validity of diagnostic tests. Chiropractors often adopt tests that they do not determine these characteristics for and need to know how to do this…”*
Haneline M [71]	1	0	Purchase	*“informs the practitioner of what is and what is not evidence-based practice”*

PMC = Article available through open access on PubMed Central.

Journal = Article available through open access on journal homepage.

Purchase = Article available for purchase from journal.

Un/Author = Unauthorized posting on Internet website.

### Articles with multiple recommendations

Six distinct articles were recommended by more than one respondent. Six respondents recommended the article, “Effectiveness of manual therapies: the UK evidence report”, by Bronfort et al. [35]. A majority of the respondents noted the comprehensive nature of this systematic review of randomized clinical trials of spinal manipulation, mobilization and massage. This article addresses varying levels of evidence and cites 322 articles on spinal manipulation and other manual therapies as treatments for musculoskeletal, headache and non-musculoskeletal conditions in an organized and easily readable manner. One respondent wrote, “This is the most comprehensive systematic review of the literature pertinent to DCs for both musculoskeletal (MSK) and non-MSK conditions”.

Two respondents each recommended five additional studies [8,36-39]. An article published in 2002 by Pickar [36], “Neurophysiological effects of spinal manipulation”, is one of the earliest publications making the list, confirming the author’s early attention to identifying the basic mechanisms of action underlying spinal manipulation and the relevance of the topic for doctors of chiropractic. One respondent wrote, “[the article] provides evidence based scientific rationale for the effects of spinal manipulation”.

Another basic science article, this one by Ianuzzi and Khalsa [39], “Comparison of human lumbar facet joint capsule strains during simulated high-velocity, low-amplitude spinal manipulation versus physiological motions,” was similarly recommended by respondents: “an important paper for mechanisms of action of spinal manipulation” and “demonstrated intrinsic biomechanical safety of lumbar spinal manipulation”.

The large population-based study by Cassidy et al. [37] “Risk of vertebrobasilar stroke and chiropractic care: results of a population-based case–control and case-crossover study”, demonstrated vertebrobasilar stroke as a very rare adverse event. This article is a relevant reference for chiropractic professionals who educate their patients, other healthcare providers and the media about the risks of chiropractic care. One respondent commented, *“This presents the best evidence about the risk of stroke after cervical manipulation”.*

A Cochrane Collaboration systematic review published by Walker and colleagues [38], “Combined chiropractic interventions for low back pain”, represented a rigorous, international, and interdisciplinary evaluation of the merits combined chiropractic therapies over spinal manipulation alone for pain and disability. Of this article, one respondent wrote, *“This systematic review shows that usual chiropractic care is as effective as many other therapies for acute or sub acute low back pain”.*

An article by Murphy et al. [8], “The establishment of a primary spine care practitioner and its benefits to health care reform in the United States,” presented a cogent health policy rationale for changing the role of chiropractors in the U.S. health care delivery system based on research evidence. Individuals interested in the evolution of the chiropractic profession should be familiar with this document, as a respondent commented, *“This article outlines the need for a primary spine care clinician in the health care system, and highlights the qualifications of DCs to fill this niche”.*

### Literature with single recommendations

Of the 41 unique PEs, 34 were unduplicated articles or books mentioned by a single respondent. Six singly recommended articles address interdisciplinary clinical decision-making for the care of patients with low back pain [40-42,50,59,72], while two texts highlight treatment approaches to neck pain and related conditions [45,55]. Five articles report findings from clinical trials of manual therapies or spinal manipulation [43,46,53,57,60], while three other papers discuss either ineffective applications, [47,65] or common side effects of manipulation [44]. Lastly, nine articles demonstrate chiropractic research priorities [30,73,74], analyze health policy issues for the chiropractic profession [14,51,69], or discuss evidence-based practice concepts for doctors and students of chiropractic [67,70,71]. That only one respondent mentioned each of these studies should not undermine their potential relevance to the chiropractic profession. Instead, many unduplicated references are important references on key concepts, evidence or issues for practicing doctors of chiropractic. For example, 10 recommended articles provide theoretical rationale or basic science evidence on the mechanisms of action of spinal or joint manipulation [48,52,56,58,61-64,66,68].

### Scholarly impact

The secondary analysis of the scholarly impact of the recommended PEs revealed interesting patterns that may further assist doctors of chiropractic in selecting from among this list of essential literature for the profession (Table 2). The Google Scholar citation analysis identified some articles as highly referenced within the field of chiropractic and in associated disciplines. Researchers have cited some chiropractic research articles more than 100 times including work by Senstad et al. [44], Hurwitz et al. [45], Pickar [36], Cassidy et al. [37], Nelson et al. [46], Bronfort et al. [35] and Olafsdottir and colleagues [47]. Several articles in fields related to chiropractic, specifically regarding the interdisciplinary care of persons with back pain, also demonstrated very high citation rates [40-43]. These articles have clearly influenced how other researchers view the field. Other articles recommended as essential literature have received much less attention from researchers. This analysis of scholarly impact does not necessarily indicate the possible influence of any article among chiropractic educators, clinicians or students.

### Article accessibility

The accessibility of scholarly articles also is an important factor for the dissemination, uptake, and clinical application of scientific knowledge. Many of the most highly cited articles were freely available as full-text articles on the Internet, either from the journal publisher or through the U.S. National Library of Medicine (NLM) and its PubMed Central (PMC) database of biomedical and life sciences literatures. For example, the articles with the broadest uptake by other authors were the low back pain clinical practice guidelines and the clinical prediction rule for identifying responders to spinal manipulation, three freely accessible articles published in *Annals of Internal Medicine* (Table 2). In contrast, articles published in *Spine*, *The Spine Journal*, and *Journal of Manipulative and Physiologic Therapeutics* were available only by subscription or direct purchase, at a cost of about $30 US per article. Perhaps not surprisingly, these articles also had lower overall citation rates from papers published in open access journals or those available from PMC. Of note, several of these articles were available on websites from individuals or institutions in possible violation of article copyright agreements.

## Discussion

This survey of chiropractic research leaders sought to identify essential literature that every doctor of chiropractic and chiropractic student should read and reference to inform evidence-based clinical practice. Survey respondents identified 41 unique articles or books they considered key readings within the field of chiropractic or related disciplines. Essential literature included basic science and clinical research, health policy statements, education-based articles, and other types of evidence for the chiropractic profession. The majority of the recommended articles (n = 34) were published in the past 10 years, with no citations prior to 1995 offered by respondents. Two or more respondents recommended six journal articles as key pieces of evidence for doctors of chiropractic and chiropractic students [8,35-39]. These articles offered evidence on the effectiveness of manual therapies [35,38], the physiological underpinnings of spinal manipulation [36,39], risks related to chiropractic care [37], and arguments for an expanded role for chiropractors within the health care system [8], all timely and important topics for the chiropractic profession. Thirty-four additional citations on a topics ranging from low back pain and neck pain, chiropractic side effects, biomechanical and physiological effects of chiropractic adjustments, research priorities, costs and access for chiropractic therapy, and evidence-based clinical practice also were identified.

Many articles selected by respondents as essential literature had achieved some degree of scholarly impact in that they were referenced by many researchers in multiple publications [35-37,40-42,46,49,50,53,54]. While the selection of scholarly literature is dependent upon the specific goals and interests of the reader, an argument is made that articles cited more often both within the profession and in disciplines with a shared scope are more essential for the chiropractic professional than articles not as widely referenced. A shared knowledge base will assist doctors of chiropractic to communicate with one another, our patients, and other healthcare professionals about the evidence underpinning various treatment approaches.

The scholarly impact and clinical importance of this essential literature for the chiropractic profession also may be influenced by the access clinicians have to scientific articles [25,32-34]. Our secondary analysis found that articles published in open access journals generally had higher citation rates than journals where article access was limited to subscribers or by purchase. Clinicians may be prevented from accessing articles recommended as essential literature by costly fees, a concern identified in previous research [75]. Editors and publishers of chiropractic and spine-related journals may wish to reconsider their access policies in order to increase use of their articles by researchers and practicing clinicians.

### Limitations

Extremely low response rate, high rate of attrition, coverage and non-response survey errors, and self-report bias are limitations of this study. This extremely low response rate and high attrition rate were problematic. While 43 of 68 potential respondents agreed to participate, only 25% completed the survey. Of these 17 respondents, eight contributed only one piece of evidence, six contributed 2–5 references, and only three contributed more than five citations. This response rate is low by survey standards [76], including among surveys of doctors of chiropractic, which average about 53% for postal surveys [77]. The reasons for the sharp decline between respondents agreeing to participate in the study and their actual participation are unknown. While only four e-mails were returned undeliverable, it is unknown how many potential respondents may have not have received the initial e-mail due to spam filters. Future studies could be designed using a multi-modal approach. The attrition rate was high as respondents could re-enter the survey at any time during deployment if they left the session after agreeing to participate. Both of these issues potentially affect the quality of data, specifically coverage and non-response error. Coverage and non-response errors result from all members of a population not having a known, nonzero chance of being included in the sample and by non-respondents potentially differing from respondents [76]. The high attrition, both in terms of starting the survey and stopping after submitting only one recommendation might be attributed to recall bias and or source amnesia (e.g., inability to remember where, when or how one has learned prior information while retaining its factual knowledge) [78]. Respondents also performed mental work to complete this short-answer survey rather than answering discrete categorical questions, which also can result in increased attrition [76]. And yet, this survey was web-based. Respondents were not asked to recall references from memory nor were they restricted from using on-line databases to identify essential literature, which might suggest either low familiarity with the chiropractic literature or a disinterest in the survey topic among respondents. Varying degrees of self-report bias also are possible. Respondents may have entered socially desired responses (i.e., often-cited references or citations from well-known researchers) or responses that might benefit themselves or their colleagues (i.e., referencing articles that either they or their colleagues have published). An additional limitation is the potential geographic bias in the survey as the potential respondents for this study were recruited from North American institutions. Future surveys should include chiropractic colleges and programs internationally. It is suspected that the philosophy and scope of chiropractic education, research, and practice differs between regions and, consequently, affect (or enhance) survey responses. Lastly, in such a rapidly progressing subject, new impactful articles have undoubtedly been published that should be included as “essential,” prior to actual publication of this study. We should disseminate thoroughly yet quickly when compiling and distributing future study results. In spite of these limitations, we consider these responses a fruitful start to this initial investigation into a previously unexplored subject, essential literature for the chiropractic profession.

### Relevance/future research

Now that a preliminary list of essential literature for doctors of chiropractic exists, a future researcher may consider surveying practicing doctors of chiropractic or chiropractic students and faculty to determine their awareness of this essential literature and to expand the key articles from the perspective of non-researchers.

## Conclusions

This survey of chiropractic research leaders resulted in a manageable list of essential literature for chiropractic students and practitioners. A variety of perspectives and values are evident when looking at the outcomes of the survey. The recommended literature might be broken down into three major categories:

1 Foundational understanding of the pathophysiology underlying chiropractic concepts and practices.

2 Importance of practitioner awareness of the state of the evidence for patient care and clinical practice.

3 Potential societal impact fostering improved integration or acceptance.

The actual impact is limited to the perspectives of the responding research leaders. Regular annual or biannual surveys could be of benefit to many in the profession.

## Abbreviations

EBCP: Evidence-based clinical practice; PE: Pieces of evidence; DC: Doctor of chiropractic; NLM: United States National Library of Medicine; PMC: PubMed Central.

## Competing interests

The authors declare that this paper is original and has not been published nor is being considered elsewhere for publication. The authors, individually and collectively, declare no conflicts of interests.

## Authors’ contributions

The corresponding author, BM, planned the design, was involved in supervision, data collection/processing, analysis/interpretation, literature search, writing, and critical review of this manuscript. JS was responsible for concept development, supervision, writing, and critical review. DD was integral for design, supervision, data collection, and writing. RB was involved in writing and supervision. SS completed the secondary analysis and assisted in interpretation, writing, and critical review of this manuscript. All authors read and approved the final manuscript.

## Authors’ information

Barbara A. Mansholt, D.C., M.S., Assistant Professor, Technique Department, Palmer College of Chiropractic, 1000 Brady St., Davenport, IA 52803, USA.

John S. Stites, D.C., Director, Community Clinics, Palmer College of Chiropractic, 2001 52nd Ave. , Moline, IL 61265, USA.

Dustin C. Derby, Ed.D., Senior Director, Institutional Planning & Research, Palmer College of Chiropractic, 723 Brady St., Davenport, IA 52803, USA.

Ronald J. Boesch, D.C., Dean of Clinics, Palmer Davenport Clinical Affairs, Palmer College of Chiropractic, 1000 Brady St., Davenport, IA 52803, USA.

Stacie A. Salsbury, Ph.D., R.N., Clinical Project Manager, Palmer Center for Chiropractic Research, Palmer College of Chiropractic, 741 Brady St., Davenport, IA 52803, USA.
